# Pelvic PET/MR attenuation correction in the image space using deep learning

**DOI:** 10.3389/fonc.2023.1220009

**Published:** 2023-08-24

**Authors:** Bendik Skarre Abrahamsen, Ingerid Skjei Knudtsen, Live Eikenes, Tone Frost Bathen, Mattijs Elschot

**Affiliations:** ^1^ Department of Circulation and Medical Imaging, Norwegian University of Science and Technology, Trondheim, Norway; ^2^ Department of Radiology and Nuclear Medicine, St. Olavs Hospital, Trondheim University Hospital, Trondheim, Norway

**Keywords:** PET/MR, attenuation correction, deep learning, prostate cancer, artificial intelligence frontiers, MRAC, pseudo-CT

## Abstract

**Introduction:**

The five-class Dixon-based PET/MR attenuation correction (AC) model, which adds bone information to the four-class model by registering major bones from a bone atlas, has been shown to be error-prone. In this study, we introduce a novel method of accounting for bone in pelvic PET/MR AC by directly predicting the errors in the PET image space caused by the lack of bone in four-class Dixon-based attenuation correction.

**Methods:**

A convolutional neural network was trained to predict the four-class AC error map relative to CT-based attenuation correction. Dixon MR images and the four-class attenuation correction *µ*-map were used as input to the models. CT and PET/MR examinations for 22 patients ([^18^F]FDG) were used for training and validation, and 17 patients were used for testing (6 [^18^F]PSMA-1007 and 11 [^68^Ga]Ga-PSMA-11). A quantitative analysis of PSMA uptake using voxel- and lesion-based error metrics was used to assess performance.

**Results:**

In the voxel-based analysis, the proposed model reduced the median root mean squared percentage error from 12.1% and 8.6% for the four- and five-class Dixon-based AC methods, respectively, to 6.2%. The median absolute percentage error in the maximum standardized uptake value (SUV_max_) in bone lesions improved from 20.0% and 7.0% for four- and five-class Dixon-based AC methods to 3.8%.

**Conclusion:**

The proposed method reduces the voxel-based error and SUV_max_ errors in bone lesions when compared to the four- and five-class Dixon-based AC models.

## 1 Introduction

The advent of prostate-specific membrane antigen (PSMA) tracers has led to the increasing adoption of PET as the modality of choice in diagnosing recurrent prostate cancer (1). For this patient group, [^68^Ga]GaPSMA-11 PET/MR has been shown to have similar diagnostic performance to [^68^Ga]Ga-PSMA-11 PET/CT in nodal and osseous metastasis (2–4) and superior performance in the detection of local recurrences due to the higher soft-tissue contrast provided by MR (2, 5). However, attenuation correction (AC), which is the most important correction required for quantitatively accurate PET imaging, remains a challenge in PET/MR imaging (6–8).

For PET/CT, the contrast of the CT images is dependent on the electron density of the imaged tissue, which in turn is related to the linear attenuation coefficient (LAC) of the PET photons (6, 9). A piecewise linear transformation of the CT Hounsfield units can be used to estimate the LAC at the PET photon energy of 511 keV (10). This approach is widely accepted as AC for PET/CT in clinical practice (6, 11). Since the signal in MR comes from proton densities and tissue relaxation times, no such straightforward relationship between the MR intensity values and LAC at the PET photon energy exists (6, 9, 11).

In current clinical practice, whole-body PET/MR AC is typically derived from Dixon MR sequences. These sequences are time-efficient to acquire and are available in all commercially available clinical PET/MR scanners (12). In four-class Dixon-based AC, Dixon MR images are segmented into four components: fat, lung, soft tissue, and background air, and each component is subsequently assigned a respective predefined LAC (13). Bone, although highly attenuating, is not accounted for in this four-class attenuation correction model. Disregarding the bone can lead to an underestimation of the standardized uptake values in and near the bone. For the pelvic region in particular, errors as large as 30% have been found in the most impacted bone lesions (14). In the staging of prostate cancer recurrence after definitive therapy, bone lesions are also fairly common and can be expected in more than 20% of the cases (15). In restaging after salvage radiotherapy, bone lesions are even more common and are observed in as many as 45% of cases (16).

The short $T2^{*}$ relaxation times and low proton density of cortical bone cause the MR signal to decay quickly in bone tissue (17) and make bone hard to distinguish from air in conventional MR images. Thus, in the four-class Dixon-based AC model, bone is classified as soft tissue. To tackle this issue, Paulus et al. (18) proposed the five-class Dixon-based AC model. This model is an atlas-based approach for whole-body PET AC based on individual registration of the major bones (skull, femur, hips, and spine, including sacrum) from a bone atlas consisting of paired Dixon MR images and bone masks. However, this method is prone to registration errors and misses bones entirely in some cases (19, 20). In a recent retrospective study of 200 patients, it was advised not to use this form of AC for PSMA PET/MR without great caution and thorough inspection of the resulting *µ*-maps (20).

Several deep learning-based approaches have also been suggested to improve AC in PET/MR. These approaches can broadly be categorized into those that only use the Dixon images (7, 21), those that require other MR sequences than the standard Dixon images (22–25), and approaches that only use the PET data (26). A different way to categorize these approaches is by whether their goal is to create a pseudo-CT image or a *µ*-map (21–23, 25, 27) or to directly correct or predict the corrected PET image itself (26).

An obvious limitation of the acquisition of additional MR sequences for AC purposes is that it requires additional scan time. Approaches that rely on the PET data itself as input data, either for *µ*-map prediction or for direct prediction of the corrected PET image itself, are dependent on the tracer for which the model is trained. These models are thus not directly applicable to multiple tracers without retraining the model.

We introduce a novel, deep learning-based approach to improving AC in pelvic PET/MR acquisitions. The proposed method aims to directly correct the errors in the PET images caused by bones not being included in the four-class AC model rather than predicting new *µ*-maps. For this purpose, a voxel-wise correction map is predicted by a convolutional neural network using Dixon MR and the four-class *µ*-map as input. The predicted correction map can subsequently be applied as a postprocessing step directly in the PET image space to correct PET images reconstructed with the four-class *µ*-map without re-reconstruction of the images. Additionally, the proposed model requires no additional sequences beyond the standard Dixon MR images and does not require retraining to be used with multiple tracers. In this work, we evaluated the quantitative impact of the proposed method on PSMA uptake in the pelvic region of patients suspected of recurrence of prostate cancer.

## 2 Materials and methods

### 2.1 Patient selection and data acquisition

This study included 49 patients who underwent same-day PET/CT and PET/MR procedures following a single tracer injection. The included cohort consisted of male patients with suspicion of lymphoma and lung cancer scanned with [^18^F]FDG (FDG), which was used for training and validation of the proposed model. A separate cohort with patients suspected of recurrence of prostate cancer after radical treatment scanned with [^68^Ga]Ga-PSMA-11 or [^18^F]PSMA-1007 was used as the test set. The PET acquisitions included one to five bed positions, where data were acquired for 5–10 min per bed. Patients with sphincter pumps and metal implants were excluded, as were those with imperfect coregistration between MR and CT.

PET and MR images were acquired on a 3T Biograph mMR PET/MR scanner (Siemens Healthineers, Erlangen, Germany, updates MR B20P and MR E11). A standard Dixon sequence was acquired for attenuation correction purposes. The scan parameters and resolution of the Dixon series varied within the dataset. The parameters of the Dixon series are summarized in **Table 1**.

**Table 1:** Scan parameters for the different Dixon series contained in the dataset.

Dixon series	Spacing (mm)	TR (ms)	TE_1_ (ms)	TE_2_ (ms)
1	2.1 × 2.1 × 3.0	3.8	1.2	1.2
2	1.3 × 1.3 × 3.0	3.8	1.2	2.5
3	2.1 × 2.1 × 2.6	3.8	1.2	2.5
4	2.6 × 2.6 × 3.1	3.6	1.2	2.5

The enumeration of the series is arbitrary. The flip angle was 10° for all variations.

Low-dose CT images were acquired at a Biograph64 PET/CT scanner (Siemens Healthineers, Erlangen, Germany) using adaptive exposure control (tube voltage: 120 kV, peak and tube current median: 34.8, range: 17–52, slice thickness: 3 mm, matrix: 512 × 512, and pixel spacing: 1.5 mm × 1.5 mm). The CT images were acquired arms-up as opposed to the MR images. The PET images from the PET/CT examination were not used in this study.

### 2.2 µ-Map generation

To generate the reference standard *µ*-map, the CT images were first registered to the Dixon MR images with the Elastix registration toolbox (28, 29) using a composite registration scheme consisting of a rigid and a deformable stage (**Supplementary Section 1**). To obtain an accurate registration between CT and MR, arms were masked out from the MR images and corresponding four-class *µ*-maps. After the registration, the CT Hounsfield unit values were scaled to their corresponding LAC at 511 keV according to parameters by Burger et al. (10).

Due to the difficulty of attaining perfect coregistration in soft tissue and bone simultaneously, only the bone information was transferred into the Dixon four-class *µ*-map from the scanner console to generate the reference standard *µ*-map image as opposed to using a scaled registered CT directly. Bone information was defined as all voxels within the CT image with a LAC of >0.1 cm^−1^. This approach is similar to Bradshaw et al. (25), who also used MR-based AC for the soft tissue classes and coregistered bone information from CT images to form the reference standard *µ*-map. The CT-to-MR coregistration of each case was closely examined, and only images found to perform well upon visual inspection were kept in the dataset. The bone information was only inserted in a mask covering the pelvic region, which was defined as all slices from approximately the upper thighs to approximately vertebrae L2 of the lumbar spine.

### 2.3 PET reconstruction

PET reconstructions were performed using the Siemens proprietary offline PET reconstruction tools (E7tools, Siemens Healthineers, Erlangen, Germany) or at the scanner console with the following reconstruction settings: ordinary Poisson ordered-subset expectation-maximization algorithm with three iterations, 21 subsets, and a 4-mm Gaussian postreconstruction filter with resolution modeling included in the reconstruction (PSF). The PET volumes were reconstructed with a 344 × 344 matrix with an in-plane resolution of 2.1 mm × 2.1 mm and a slice thickness of 2.0 mm. Absolute scaling (30) was used for scatter correction in patients imaged with [^68^Ga]Ga-PSMA-11, and relative scaling was used for [^18^F]PSMA-1007 images and FDG images (31).

### 2.4 Correction method

A deep convolutional neural network was trained to predict the correction map between the PET images reconstructed using the reference standard *µ*-map, referred to as PET_CT+MR_, and the PET images reconstructed using the Dixon-based four-class *µ*-map, PET_4C_. The correction map was calculated as shown in Eq (1) For more details, see **Supplementary Section 2**.


(1) $$\text{Correction map }=\frac{{\text{PET}}_{4\text{C}}-{\text{PET}}_{\text{CT}+\text{MR}}}{{\text{PET}}_{4\text{C}}}$$

The correction map was only estimated within the mask covering the pelvic region. Dixon in-phase, Dixon out-of-phase, and the four-class Dixon-based *µ*-map were used as inputs to the network. The images were resampled to the PET image space and min–max scaled to intensities in the range [0,1]. A threshold intensity mask was used to set intensity values outside the body contour to zero.

**Figure 1:**
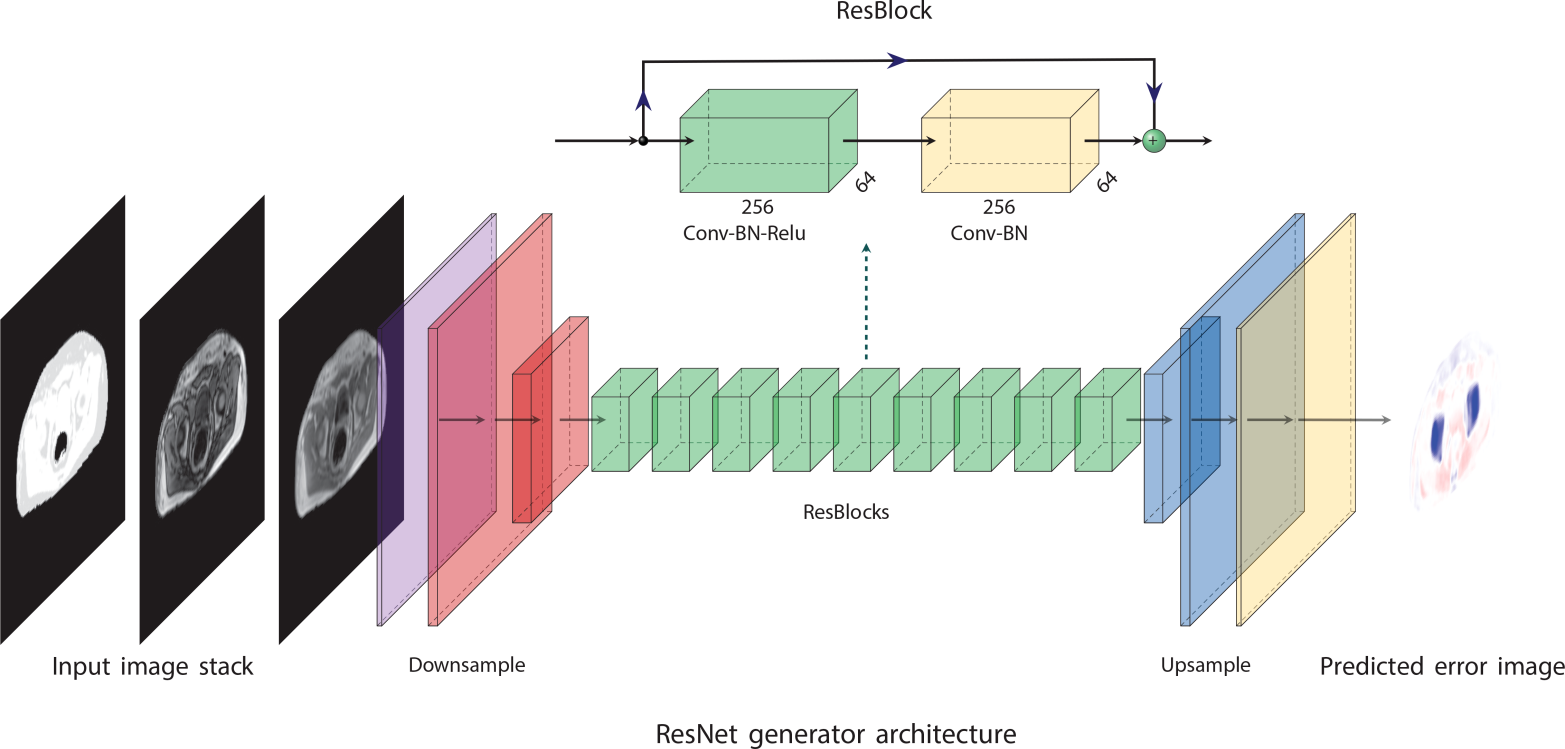
The network architecture is based on ideas proposed by Johnson et al. (32). It consists of nine residual blocks (33) (ResBlocks) between a convolutional front-end and a transposed convolutional back-end. The convolutional front-end downsamples the images to one-fourth of their original resolution, and the transpose convolutional back-end upsamples the images to their original resolution. The figure was made using PlotNeuralNet (34).

An overview of the network architecture is given in **Figure 1**. The network consisted of a nine-block ResNet (33) architecture inspired by Johnson et al. (32) and Wang et al. (35). The network was trained using *L*2 loss and an Adam optimizer (36) for 100 epochs with a linearly decaying learning rate starting from epoch 50. The code for training the network and the hyperparameters used in the final model is available on github (https://github.com/ntnu-mr-cancer/PETMR-4CMRAC-Correction-maps).

**Figure 2:**
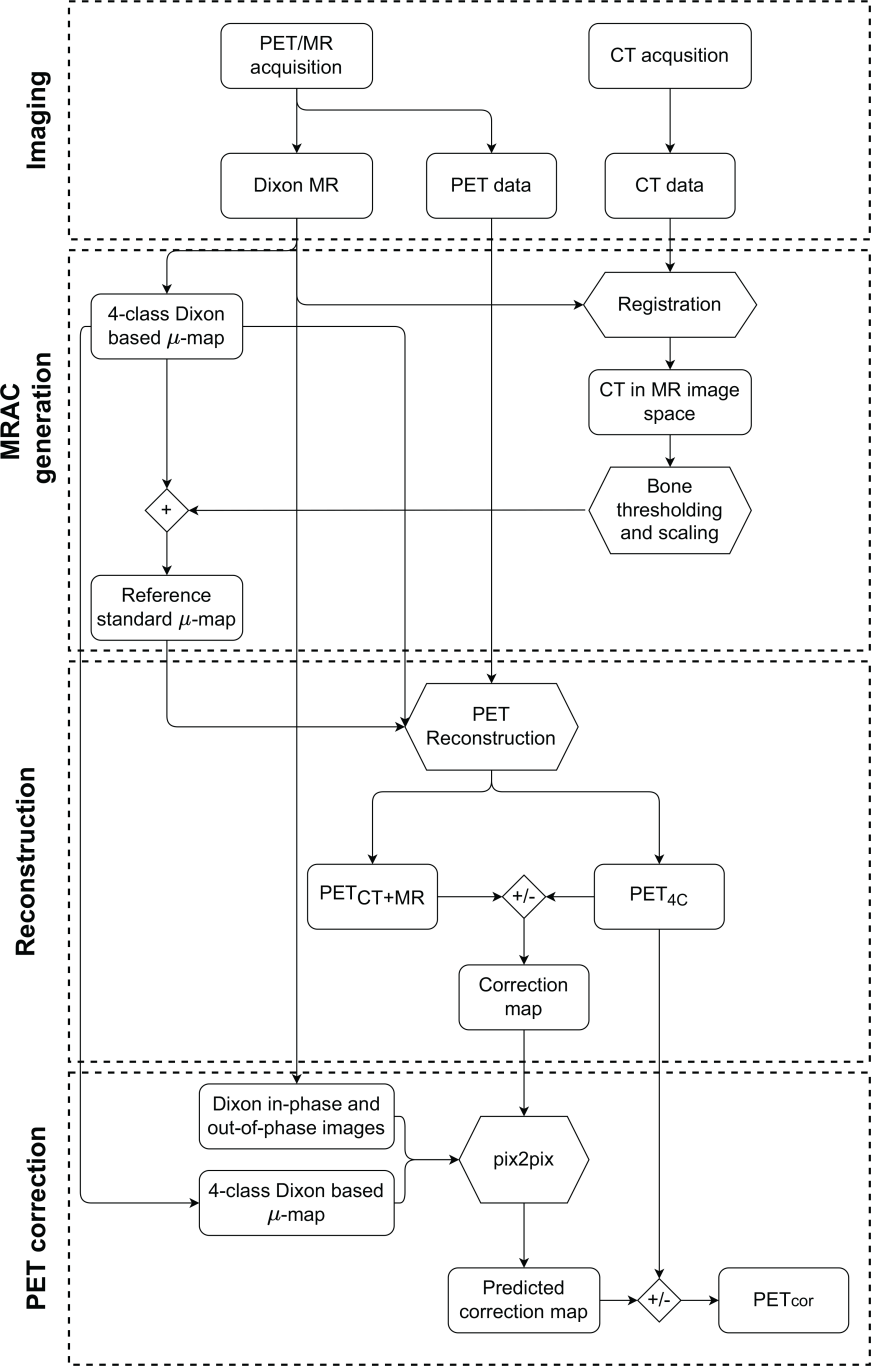
Graphical overview of the methodology from acquisition to generation of the corrected PET image (PET_cor_). The node labeled + refers to overwriting linear attenuation coefficients (LAC) in the four-class Dixon-based *µ*-map with CT bone information scaled to LAC at 511 keV. The nodes labeled +/-represent the creation of the correction map as specified in Eq (1), and the application of the correction map to PET reconstructed with the four-class Dixon-based *µ*-map (PET_4C_). PET_MR+CT_ is PET reconstructed using reference standard *µ*-map, which is obtained by using a four-class Dixon-based *µ*-map for soft tissue and bone information from a co-registered CT image.

Results for additional tested network architectures can be found in **Supplementary Section 3**. The output of the network is a predicted correction map, which is used to correct the PET_4C_ images by solving Eq (1). for PET_CT+MR_. The resulting corrected PET image will be referred to as PET_cor_. An overview of the methods from image acquisition to corrected PET images is given in **Figure 2**.

### 2.5 Analysis and statistics

Voxel- and lesion-based analysis was performed to assess the performance of the method. For the voxel-based analysis, only voxels within the pelvic mask that had an activity concentration of *>* 300 Bq ml^−1^ were used in the calculation. The relative error image (RE*_x_*), defined as shown in Eq. (2),ResNet generator architecture


(2) $$RE_{x}\;=\frac{I_{x}-I_{GS}}{I_{GS}}$$

Where *I* is the image intensity, GS represents the reference standard PET image PET_CT+MR_, and *x* is either PET image that is compared to the PET_CT+MR_ (i.e., PET_cor_ or PET_4C_). RE_x_ was used as a basis to calculate the mean absolute percentage error (MAPE) and root-mean-squared percentage error (RMSPE) that were used as quantitative metrics in the voxel-based analysis. MAPE and RMSPE were defined as shown in Eq. (3)


(3) $$\begin{array}{l} MAPE\;=\frac{1}{n_{\text{mask}}}\sum_{i\in mask}\left\|RE_{x}\right\|\\ \text{RMSPE}=\sqrt{\mu_{{\text{RE}}_{x}}^{2}+\sigma_{{\text{RE}}_{x}}^{2}}, \end{array}$$

Where *µ*_RE_ *_x_* and *σ*_RE_ *_x_* are the mean and standard deviation, RE*_x_* and *n*_mask_ are the number of voxels within the mask that satisfy the activity concentration threshold. It is understood that the summation in the definition of MAPE and the summations performed in calculating *µ*_RE_ *_x_* and *σ*_RE_ *_x_* in RMSPE are performed only over *n*_mask_.

To assess lesion performance, lesions were extracted from radiology reports. The lesion performance was measured as the relative error and MAPE of the maximum standardized uptake values (SUV_max_) of the corresponding lesions between the PET images.

All presented values are given as medians with ranges in brackets unless otherwise mentioned. A two-sided Wilcoxon signed-rank test was performed to assess whether there were any differences between RMSPE values of the PET_cor_ image and PET images reconstructed using four- and five-class Dixon-based AC (PET_4C_ and PET_5C_) in the test set. A test of difference was also made for each of the tracers in the test set separately. Benjamini–Hochberg correction was used to correct the *p*-value for multiple comparisons where applicable (37). A Mann–Whitney *U* test was used to compare differences between the RMSPE of [^68^Ga]Ga-PSMA-11 and [^18^F]PSMA-1007 images. No statistical tests were performed for the lesion-based analysis due to the limited number of samples. A difference was considered significant if *p <* 0.05 was achieved.

## 3 Results

From the 49 included patients, two patients were removed from the dataset due to severe artifacts in the PET images and eight were removed due to suboptimal coregistration between CT and MR. This resulted in a training set consisting of 18 patients scanned with FDG, a validation set of four patients scanned with FDG, and a test set consisting of 17 patients scanned with [^68^Ga]Ga-PSMA-11 or [^18^F]PSMA-1007. An overview of the dataset is presented in **Table 2**, and a flow diagram of patient inclusion can be found in the **Supplementary Material (S1)**. From radiology reports of patients in the test set, 16 soft tissue lesions and four bone lesions were extracted.

**Table 2:** Summary statistics for included patients.

Fold	Train	Val	Test	Test
Tracer	FDG (*n* = 18)	FDG (*n* = 4)	18F-PSMA (*n* = 6)	68Ga-PSMA (*n* = 11)
PI (min)	98 [88–157]	116 [90–168]	136 [116–165]	58 [48–105]
Weight (kg)	88 [71–120]	82 [73–92]	78 [62–86]	85 [74–103]
Dose (MBq)	352 [280–478]	328 [290–368]	200 [153–218]	149 [133–157]

Data is given as median values with ranges in brackets. Fold specifies which part of the data was used for training (Train), validation (Val) and testing (Test) of the model. PI = time between tracer injection and image acquisition.

**Figure 3:**
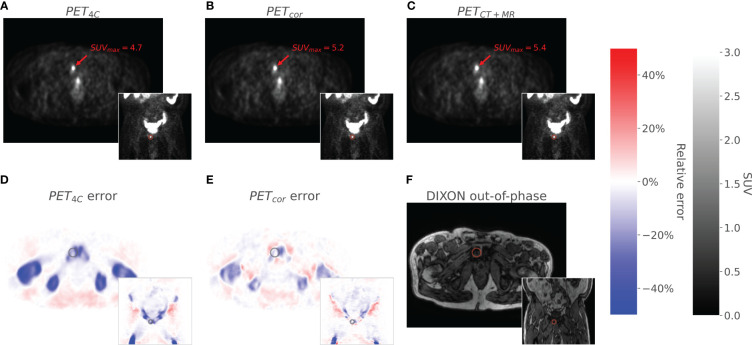
The figure shows axial and coronal images of PET_4C_ **(A)**, PET_cor_ **(B)**, and PET_CT_ **(C)**. The PET_4C_ error **(D)** is the relative error image between the PET_4C_ image and PET_MR+CT_, and the PET_cor_ error **(E)** is the relative error image between PET_cor_ and PET_MR+CT_. Values outside the body contour in the relative error images are set to zero. The Dixon out-of-phase image is given as an anatomical correlate **(F)**. A lesion located in the *os pubis* is highlighted in all images.

Training the convolutional neural network took approximately 4 h on a single NVIDIA GeForce GTX 1080 Ti. An example of a corrected PET image can be seen in **Figure 3**. The corrected PET images were found to closely resemble the reference standard PET_CT+MR_ images.

**Figure 4:**
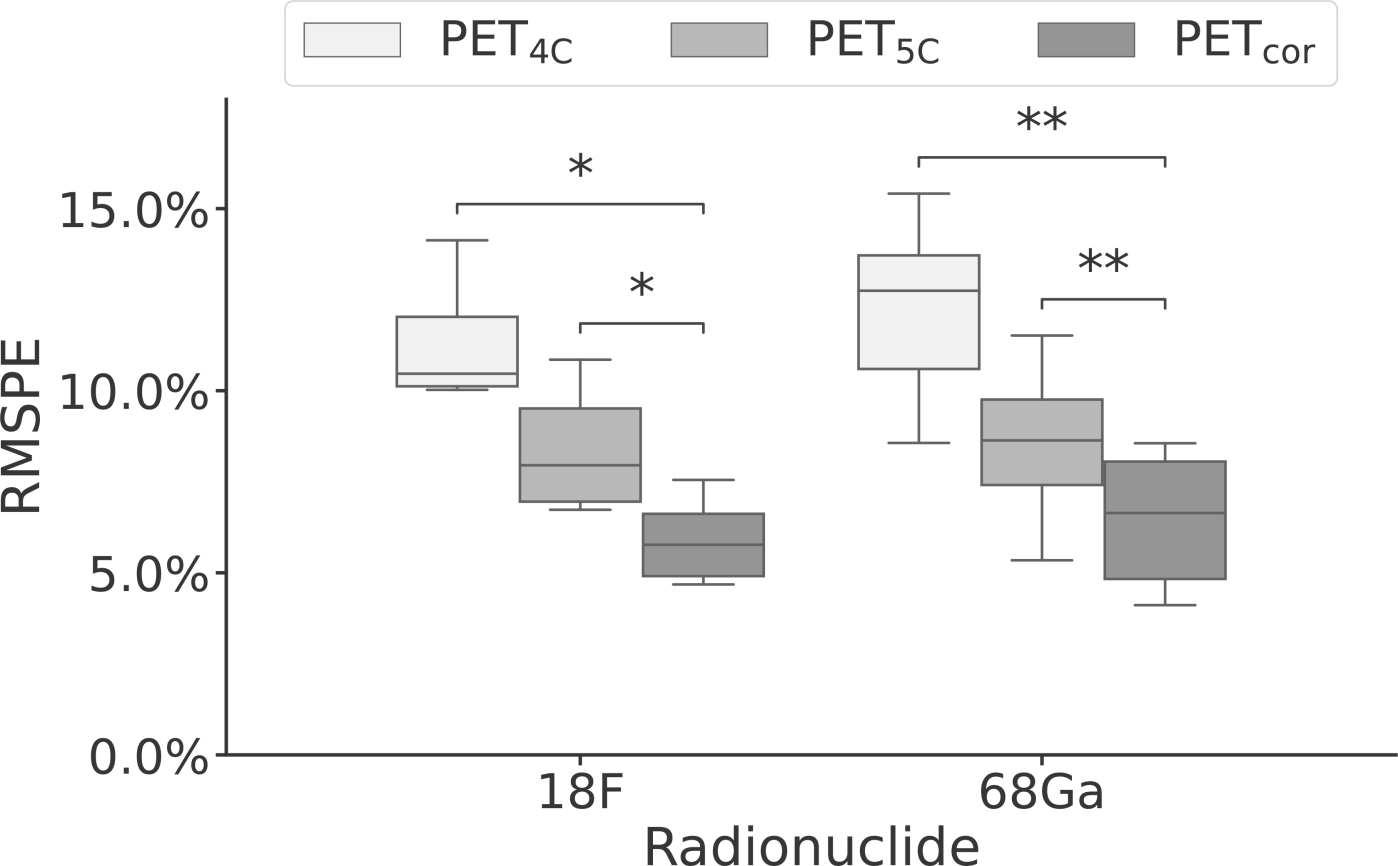
Box-and-whisker plot of the root mean squared percentage error performance stratified by radionuclide for PET reconstructed using four- and five-class Dixon-based attenuation correction (PET_4C_ and PET_5C_) and the proposed model (PET_cor_). The central line within each box is the median. The box edges extend from the 25th to the 75th percentile. Values were considered outliers if they were more than 1.5 times the interquartile ranges of the box edges. The whiskers extend to the most extreme nonoutlier value in the data. ^*^ *p <* 0.05; ^**^ *p <* 0.01.

The voxel-based RMSPE and MAPE were 12.1% [8.6%, 15.4%] and 6.2% [4.0%, 10.3%], respectively, for the PET_4C_ images and 8.6% [5.3%, 11.5%] and 3.5% [2.3%, 5.1%], respectively, for the PET_5C_ images. In the PET_cor_ images, the RMSPE was 6.2% [4.1%, 8.6%] and the MAPE was 3.3% [2.3%, 4.6%]. The error in PET_cor_ is thus approximately reduced by half compared to the PET_4C_ images. A significant difference was found between the RMSPE of the PET_cor_ images and the PET_4C_ images (*p <* 0.0001) and between PET_cor_ images and the PET_5C_ images (*p <* 0.0001). A significant difference was also found between the RMSPE values for [^68^Ga]Ga-PSMA-11 and [^18^F]PSMA-1007 patients individually between PET_cor_ and both PET_4C_ and PET_5C_, as shown in **Figure 4**. No significant difference was found when comparing the RMSPE of PET_cor_ between patients acquired with [^68^Ga]Ga-PSMA-11 and [^18^F]PSMA-1007.

**Figure 5:**
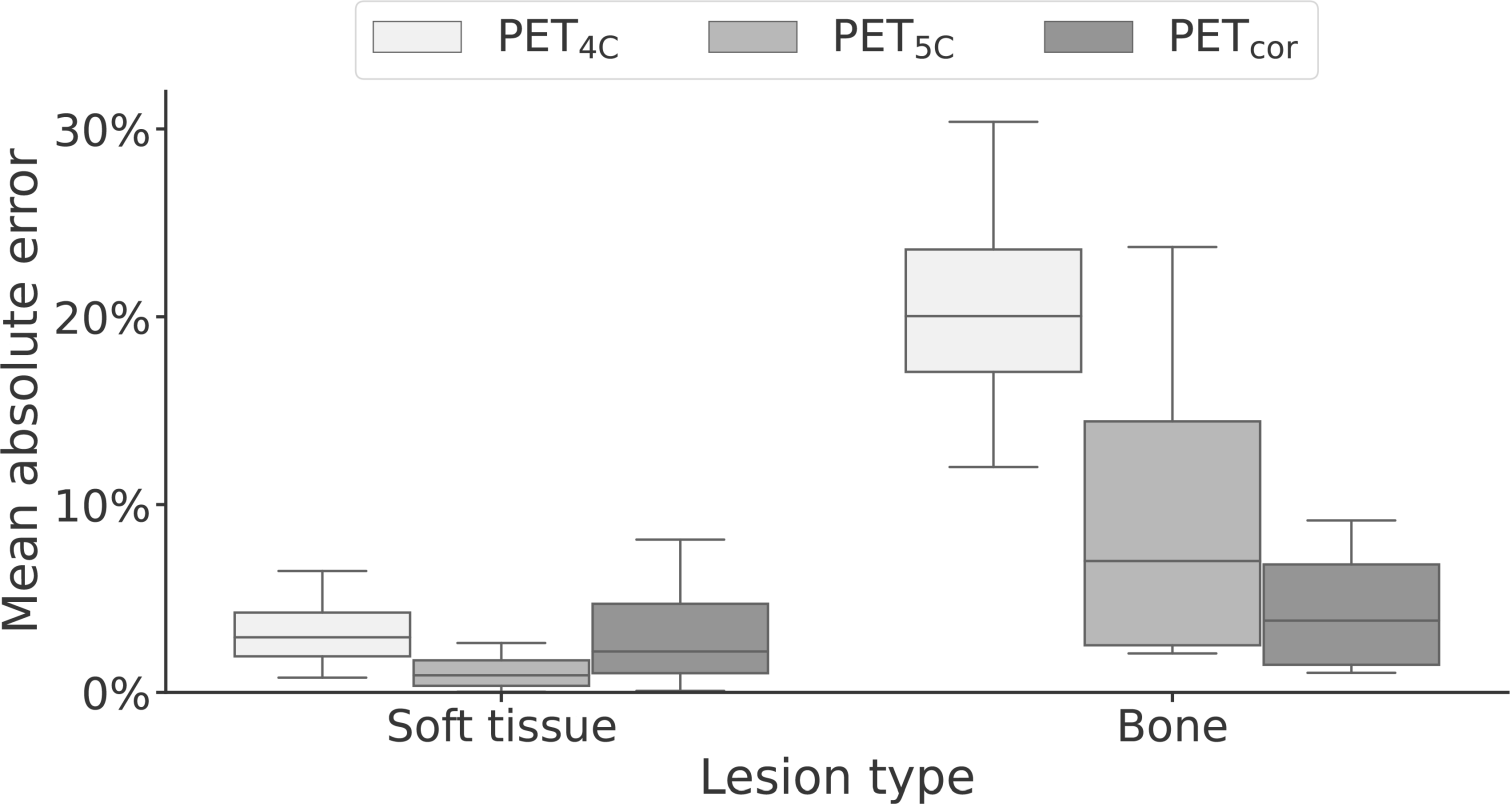
Box-and-whisker plot of the mean absolute error of soft-tissue and bone lesions for PET reconstructed using four- and five-class Dixon-based attenuation correction (PET_4C_ and PET_5C_) and the proposed model (PET_cor_). The central line within each box is the median. The box edges extend from the 25th to the 75th percentile. Values were considered outliers if they were more than 1.5 times the interquartile ranges of the box edges. The whiskers extend to the most extreme nonoutlier value in the data.

The lesion performance is summarized in **Table 3** and **Figure 5**. Performance in soft tissue lesions improved marginally from a MAPE of 2.9% [0.8%, 6.5%] in PET_4C_ to 2.2% [0.1%, 8.1%] for PET_cor_. For bone lesions, we observed more than a fivefold decrease in MAPE from 20.0% [12.0%, 30.4%] in PET_4C_ to 3.8% [1.0%, 9.2%] in PET_cor_. PET_5C_, in comparison to the proposed model, had lower MAPE in soft tissue lesions and higher MAPE in bone lesions.

**Table 3:** Lesion performance of PET images reconstructed using four- and five-class Dixon-based *µ*-maps (PET_4C_ and PET_5C_) and PET corrected using the proposed model (PET_cor_) relative to the reference standard.

	Type	Bone (*n* = 4)	Soft tissue (*n* = 16)
Model	Error		
4-class	Absolute percentage error	20.0% [12.0%, 30.4%]	2.9% [0.8%, 6.5%]
	Relative error	−20.0% [−30.4%, − 12.0%]	−2.7% [−6.5%, 6.4%]
5-class	Absolute percentage error	7.0% [2.1%, 23.7%]	0.9% [0.0%, 2.6%]
	Relative error	−7.0% [−23.7%, −2.1%]	−0.5% [−2.6%, 2.5%]
Corrected	Absolute percentage error	3.8% [1.0%, 9.2%]	2.2% [0.1%, 8.1%]
	Relative error	1.3% [−6.0%, 9.2%]	−1.9% [−8.1%, 4.4%]

Statistics are given as medians, with ranges in brackets.

We observed a decrease in performance with increasing postinjection time (PI) in the test set. In a simple linear regression model, a significant linear trend (*p <* 0.05, *r* ^2^ = 0.30) was found between the voxel-based RMSPE measurements and the PI time (see **Supplementary Section 5** for further details).

## 4 Discussion

In this study, we propose a novel attenuation correction method that seeks to directly correct for the errors obtained by not including bone when using the conventional four-class Dixon-based AC. We show that the model reduces quantification errors in a voxel-based analysis and in bone lesions compared to four- and five-class Dixon-based methods. For soft tissue lesions, the performance remains similar to that of the four-class Dixon-based AC model.

Our method can be directly applied as a correction filter in the image space to PET_4C_ images without the need for additional reconstruction or the acquisition of additional MR sequences. It can thus also be used to correct PET_4C_ images retrospectively, as long as Dixon MR images are available. This distinguishes it from other models that seek to improve pseudo-CT generation. The model also only relies on Dixon MR images, which are fast to acquire. Furthermore, like the models that predict pseudo-CT images from MR series (21–23, 25, 27), but unlike the models that predict the pseudo-CT images or AC and scatter-corrected PET directly from non-attenuation corrected PET (26, 38, 39), the predicted correction maps are not tracer-dependent.

We observed that the proposed method is robust to variations in tracer type and scatter correction method. The training and validation sets consisted of FDG images, whereas the test set consisted of [^68^Ga]Ga-PSMA11 and [^18^F]PSMA-1007 images. Since there were no [^68^Ga]Ga-PSMA-11 images in the training data, the model had only seen images reconstructed using relative scaling during scatter correction in training. Nevertheless, no differences in performance were seen between [^68^Ga]Ga-PSMA-11 and [^18^F]PSMA-1007 patients in the test set.

A decrease in performance was seen with increasing PI. This trend can be explained by the higher number of counts acquired at lower PI, making the correction map that the model tries to predict less noisy. All patients had PET/MR and PET/CT acquisition performed on the same day. For around half of the patients in the test set, the PET/CT images were acquired at what was considered the optimal PI for the given tracer, and the PET/MR images were acquired once the PET/CT examination was finished. If all PET/MR had been acquired closer to the recommended PI, we would expect to see a slight increase in performance.

Multiple other methods have been suggested for improving PET/MR attenuation correction in the pelvic region (7, 22, 23, 25, 27). Bradshaw et al. (25) proposed a model based on the Deep Medic CNN architecture (40). In this model, T2 and T1 Lava Flex images were used for the prediction of pseudo-CT images, resulting in an RMSPE of 4.9% in the reconstructed PET image. Leynes et al. (23) used Dixon-based images and zero-echo-time images as input to a deep learning model based on the UNET architecture to generate *µ*-maps. The resulting PET reconstruction had an RMSPE of 2.85%. Similar to Bradshaw et al. (25), Torrado-Carvajal et al. (7) used a UNET-like architecture to create a pseudo-CT image using solely Dixon MR images as input. Their approach resulted in an absolute mean relative change of 1.83%.

A different family of models is composed of models that use non-attenuation corrected PET images as input and either the pseudo-CT (41) or the attenuation and scatter-corrected PET as output (26, 38). Though good performance can be obtained with these models, they are tracer-dependent and would likely need to be retrained for optimal performance with each new tracer. In addition, the models proposed in the literature (26, 38, 41) have only been trained and validated on PET/CT data so far, and their performance is yet to be assessed using data acquired on the PET/MR system.

Though many methods have been proposed, it is difficult to directly compare their performance. As pointed out by Lee (42), there is substantial heterogeneity in the choice of PET reconstruction parameters and performance metrics. There is also no reference dataset that can be used to benchmark the performance of different models. What is considered gold standard AC also varies between studies. In this study, we directly estimate the error of not including bones in the four-class Dixon-based *µ*-maps. The most common is to use registered CT images translated to LAC at 511 keV as the gold standard *µ*-map. This does, however, rely on a close-to-perfect coregistration between CT and MR images, which can be difficult to obtain in practice (25, 43, 44). We adopted a method similar to Bradshaw et al. (25) in which soft-tissue classes and air in the *µ*-map are derived from the MR images, and bone and osseous tissues are derived from the CT images.

A primary limitation of this study is the limited number of patients. The training set consisted of only 18 patients, and the test set consisted of 17 patients. Since the model was trained using a 2D network, this still constituted a considerable number of images, but we do not expect that the limited training set was able to capture all the expected interpatient variability. The number of lesions was also limited. In bone, where the model had the largest impact in our testing, only four lesions were found.

The current method is also limited to pelvic imaging only. Adapting to a different clinical application would require retraining of the model. Additionally, in the current work, only a limited subset of reconstruction parameters was used, and we did not evaluate the robustness of the method toward changes in reconstruction parameters. However, since the method is trained to predict correction maps for the PET images directly, we suspect the model to be subject to similar variability between reconstruction parameters as SUV_max_ measurements themselves (45, 46). Lastly, the model is only tested on a specific patient cohort consisting of elderly male patients. Consequently, the model must be evaluated on a larger and more diverse patient cohort before implementation in clinical practice is justified.

## 5 Conclusion

Direct correction of four-class Dixon-based AC PET in the image space is a viable method for improving AC of pelvic PSMA PET/MR imaging. The method is tracer-independent, requires only the Dixon MR series and the four-class Dixon-based *µ*-map, and can be retrospectively applied to PET data without the need for re-reconstruction. It gives superior performance to the four- and five-class Dixon-based AC in a voxel-based RMSPE analysis and for quantification of bone lesion uptake.

## Data availability statement

The datasets presented in this article are not readily available because The data are not publicly available because they contain information that could compromise research participant privacy/consent. Requests to access the datasets should be directed to bendik.s.abrahamsen@ntnu.no.

## Ethics statement

The studies involving humans were approved by Regional Committee for Medical and Health Research Ethics Mid Norway (FDG data: identifier REK2014/1289 and PSMA data: identifier REK2020/83009). The studies were conducted in accordance with the local legislation and institutional requirements. The participants provided their written informed consent to participate in this study.

## Author contributions

All authors contributed to the study conception and design. The data collection of the FDG lymphoma and lung cancer dataset was performed by LE. Data collection of the [18F]PSMA-1007 and [68Ga]Ga-PSMA-11 images was performed by IK and BA. Analysis and interpretation of data was performed by ME, TB and BA. The first draft of the manuscript was written by BA and all authors commented on previous versions of the manuscript. All authors read and approved the final manuscript.
