# Supplementary material for: Pelvic PET/MR attenuation correction in the image space using deep learning
Source: Front Oncol. 2023 Aug 24;13:1220009. doi: 10.3389/fonc.2023.1220009 (PMC10484800; doi:10.3389/fonc.2023.1220009)
Supplement: Supplementary file 1 [file DataSheet_1.pdf]

Supplementary Material

1 REGISTRATION PARAMETERS

Co-registrations were performed using *elastix* (v5.0.1) [1].

1.1 Registration call

```
elastix -f "in-phase_DIXON_path"\
    -m "CT_path"\
    -out "out_path"\
    -p "translation_parameter_file"\
    -p "deformable_parameter_file"\
    -fMask "MR_body_contour_mask_path"\
    -mMask "CT_body_contour_mask_path"
```

1.2 Translation stage parameters

```
(Registration "MultiResolutionRegistration")
(Metric "AdvancedMattesMutualInformation")
(ImageSampler "RandomCoordinate")
(Interpolator "LinearInterpolator")
(ResampleInterpolator "FinalBSplineInterpolator")
(Resampler "DefaultResampler")
(Optimizer "AdaptiveStochasticGradientDescent")
(FixedImagePyramid "FixedSmoothingImagePyramid")
(MovingImagePyramid "MovingSmoothingImagePyramid")

// ********** ImageTypes
(FixedInternalImagePixelType "float")
(MovingInternalImagePixelType "float")
(UseDirectionCosines "true")

// ********** Pyramid and optimizer
(NumberOfResolutions 3)
(MaximumNumberOfIterations 300 300 300)

// ********** Transform
(Transform TranslationTransform)

// Initialization
(AutomaticTransformInitialization "true")
(AutomaticTransformInitializationMethod "GeometricalCenter")

// ********** Mask options
(ErodeFixedMask "true")
(ErodeMovingMask "true")

// ********** Result options
```

```
(ResultImageFormat "nii")
(WriteResultImage "true")
(DefaultPixelValue -1000.000000)
(ResultImagePixelType "float")

// ********** Combination of Transforms
(HowToCombineTransforms "Compose")
```

1.3 Deformable stage parameters

```
(Registration "MultiMetricMultiResolutionRegistration")
(ImageSampler "RandomCoordinate")
(Interpolator "LinearInterpolator")
(ResampleInterpolator "FinalBSplineInterpolator")
(Optimizer "AdaptiveStochasticGradientDescent")
(FixedImagePyramid "FixedSmoothingImagePyramid")
(MovingImagePyramid "MovingSmoothingImagePyramid")

(Resampler "DefaultResampler")

// ********** ImageTypes
(FixedInternalImagePixelType "float")
(MovingInternalImagePixelType "float")
(UseDirectionCosines "true")

// ********** Pyramid and optimizer
(NewSamplesEveryIteration "true")
(NumberOfSpatialSamples 10240)
(NumberOfJacobianMeasurements 20000 40000 80000 80000)

(NumberOfResolutions 4)
(FinalGridSpacingInVoxels 32.0 32.0 32.0)
(GridSpacingSchedule 6.0 6.0 6.0 4.0 4.0 4.0 2.5 2.5 2.5 1.0 1.0 1.0)

(MaximumNumberOfIterations 500 500 500 500)

// ********** Transform
(Transform "BSplineTransform")

// ********** Metric
(Metric "AdvancedMattesMutualInformation" "TransformBendingEnergyPenalty")

(Metric0Weight 1)
(Metric1Weight 1)

// Initialization
(AutomaticTransformInitialization "true")
(AutomaticTransformInitializationMethod "GeometricalCenter")
```

```
// ********** Mask options
(ErodeFixedMask "true")
(ErodeMovingMask "true")

// ********** Result options
(ResultImageFormat ".mha")
(ResultImagePixelType "short")
(WriteResultImage "true")
(DefaultPixelValue -1000.000000)

// ********** Combination of Transforms
(HowToCombineTransforms "Compose")
```

2 CORRECTION MAP DEFINITION

In this appendix, the hat symbol ($\widehat{\ }$) is used to denote estimated values. The correction map, as defined in equation (S1), was chosen because it was more numerically stable than the standard bias definition when calculating the corrected PET image (PET_{cor}, or equivalently $\widehat{\text{PET}_{\text{CT+MR}}}$), e.g. the PET image where we have applied the predicted correction map. The correction of PET reconstructed using the 4-class Dixon-based attenuation correction (PET_{4C}), can with this definition be performed using equation (S2).

$$\text{Correction map} = \frac{\text{PET}_{\text{4C}} - \text{PET}_{\text{CT+MR}}}{\text{PET}_{\text{4C}}} \tag{S1}$$

$$\text{PET}_{\text{cor}} = \widehat{\text{PET}_{\text{CT+MR}}} = \text{PET}_{\text{4C}} - \widehat{\text{Correction map}} \cdot \text{PET}_{\text{4C}} \tag{S2}$$

If we would rather use the conventional definition of *bias* where the ground truth is in the denominator (equation S3)

$$\text{Bias} = \frac{\text{PET}_{\text{CT+MR}} - \text{PET}_{\text{4C}}}{\text{PET}_{\text{CT+MR}}} \tag{S3}$$

we would have to use equation S4 to obtain the corrected PET image.

$$\text{PET}_{\text{cor}} = \widehat{\text{PET}_{\text{CT+MR}}} = \frac{\text{PET}_{\text{4C}}}{1 - \widehat{\text{Bias}}}. \tag{S4}$$

Equation (S4), will as $\widehat{\text{Bias}} \rightarrow 1, \widehat{\text{PET}_{\text{CT+MR}}} \rightarrow \infty$. By using the the correction map as defined in equation (S1) we avoid the possibility of dividing by zero when calculating $\widehat{\text{PET}_{\text{CT+MR}}}$. These issues can be prominent is the air regions outside the body contour of the patients, where relative error between reconstruction can become very large.

3 TESTED NETWORKS

Table 3 gives and overview of tested networks in the work. The networks can be divided in two groups, pix2pix generative adversarial networks [2] and convolutional neural networks. A full overview of the hyperparameters used to train each network can be found on github (`https://github.com/ntnu-mr-cancer/PETMR-4CMRAC-Correction-maps`).

Model	Model family	RMSPE	MAPE
**ResNet 9 blocks**	**CNN**	**6.2% [4.1%, 8.6%]**	**3.3% [2.3%, 4.6%]**
ResNet 12 blocks	CNN	6.4% [4.2%, 8.7%]	3.2% [2.4%, 4.8%]
Squeeze-and-Excitation ResNet 9 blocks [3]	CNN	6.2% [4.2%, 8.5%]	3.2% [2.4%, 4.7%]
UNET	CNN	7.1% [4.7%, 9.9%]	4.0% [3.0%, 5.4%]
Pix2pix 9 block ResNet	Pix2pix	6.5% [4.4%, 8.6%]	3.5% [2.5%, 4.7%]
Pix2pix UNET	Pix2pix	7.0% [4.8%, 9.8%]	3.9% [2.9%, 5.4%]
4-class	Base	12.1% [8.6%, 15.4%]	6.2% [4.0%, 10.3%]
5-class	Base	8.6% [5.3%, 11.5%]	3.5% [2.3%, 5.1%]

Table S1. Performance overview of tested networks. The results of 4-class and 5-class dixon based attenuation correction is included for reference. The selected network is highlighted is bold. The performance is given as median with ranges in brackets. MAPE = mean absolute percentage error, RMSPE = root mean squared percentage error, CNN = convolutional neural network.

4 PATIENT INCLUSION

Figure S1 shows a flow diagram of patient inclusion. From the 49 included patients, 2 were excluded due to image artifact and 8 were excluded due to sub-optimal registration between CT and MR. The implementation of the final model does not rely on image co-registration, only the creation of the dataset used to train it does. Good registration was defined as having a maximum surface to surface distance in bone of no more than 5mm upon visual inspection. The most common misalignment in this dataset was due to registration errors in the greater trochanter. This was likely caused by the fact that PET/CT images were acquired legs extended as opposed to PET/MR images that were acquired with knees bent as is clinical routine at out institution.

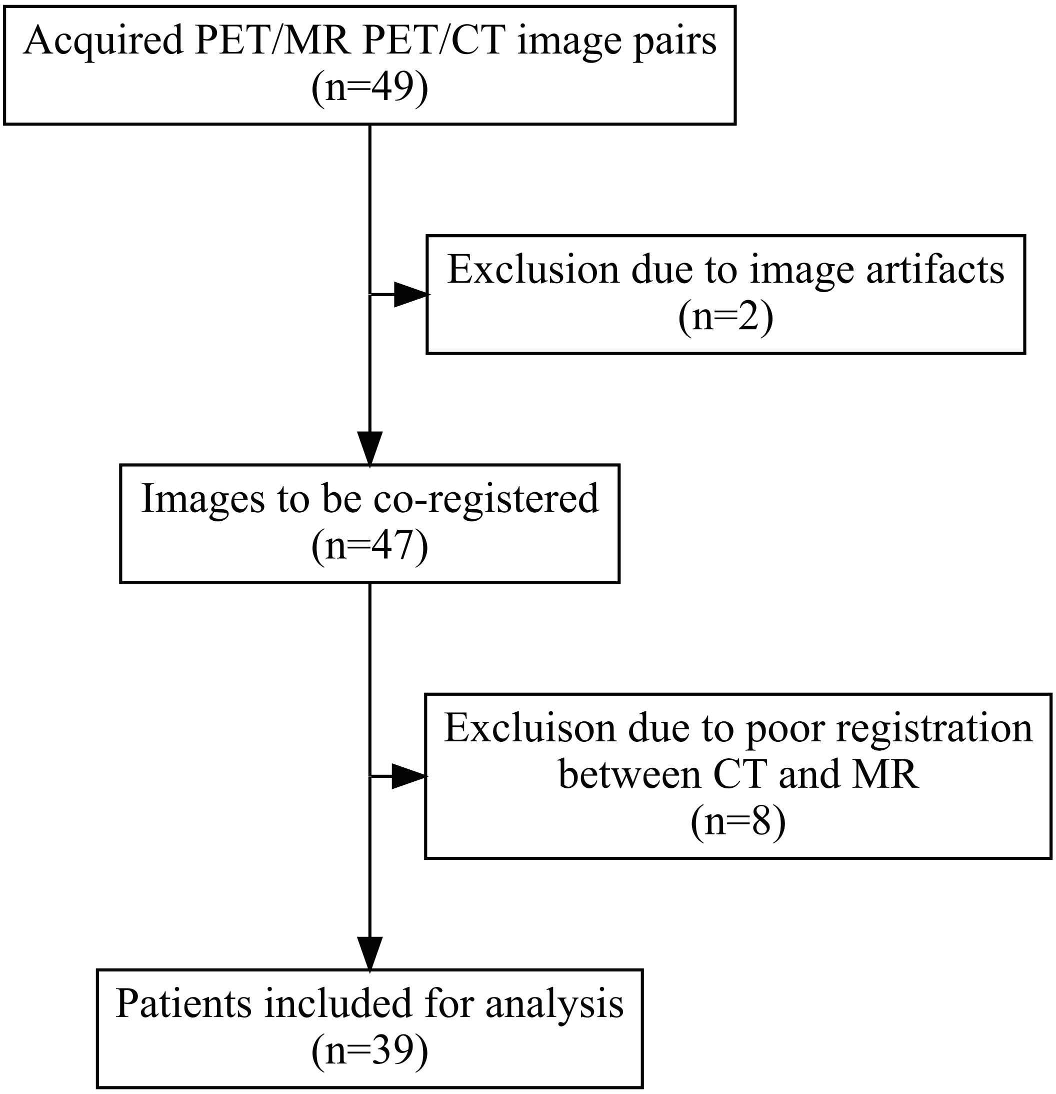

Figure S1. Flow diagram of patient inclusion. 49 patients matched inclusion criteria. 2 patients were removed due to image artifacts; one due to severe halo artifacts in the PET image data the other due to a coordinate shift of reconstructed PET data with respect to μ-map. 8 patients were dropped due to sub-optimal registration between CT and MR.

5 FACTORS IMPACTING PERFORMANCE

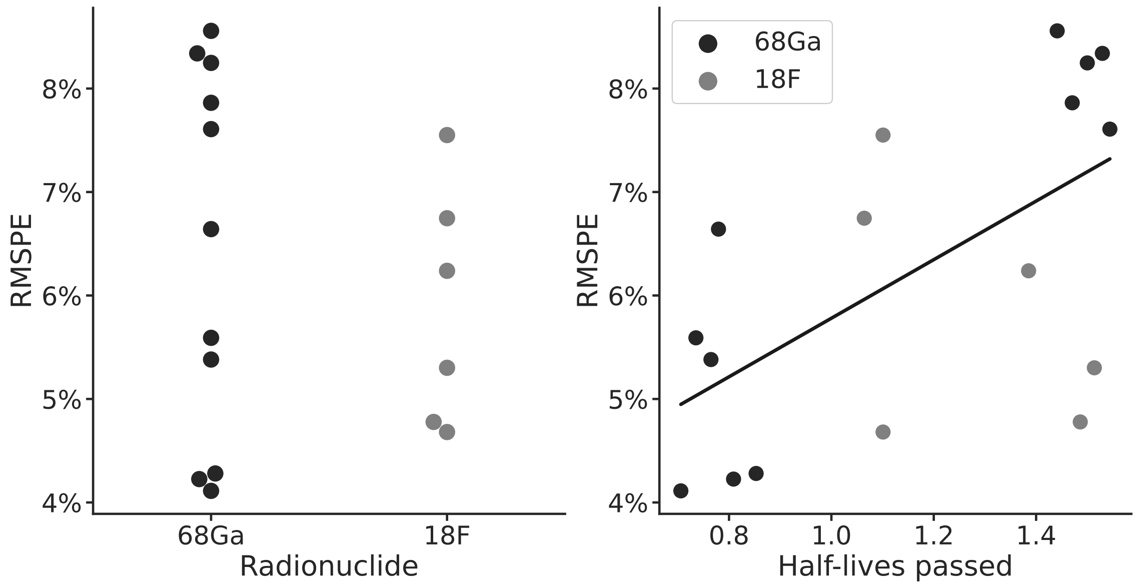

Figure S2. Performance measured as root mean squared percentage error (RMSPE) as a plotted against nucleotide (18F = ^{18}F-PSMA, 68Ga = ^{68}Ga-PSMA) and half-life. There is a linear trend between increasing the number of half-lives passed and the measured RMSPE.

Table S2. Results for a simple linear regression of root mean squared percentage error against half-lives passed. CI = confidence interval.

variable	Coefficient	p	Adjusted r^2	95% CI
Intercept	2.95%	0.024	0.318	0.45% - 5.45%
Half-lives passed	2.83%	0.011	0.318	0.76% - 4.90%

REFERENCES

[1]S. Klein, M. Staring, K. Murphy, M. A. Viergever, and J. P. W. Pluim. elastix: A toolbox for intensity-based medical image registration. *IEEE Trans. Med. Imaging*, 29(1):196–205, 2010.

[2]Phillip Isola, Jun-Yan Zhu, Tinghui Zhou, and Alexei A. Efros. Image-to-image translation with conditional adversarial networks. In *Proceedings of the IEEE Conference on Computer Vision and Pattern Recognition (CVPR)*, July 2017.

[3]Jie Hu, Li Shen, Samuel Albanie, Gang Sun, and Enhua Wu. Squeeze-and-excitation networks, 2019.
